# Editorial

**Published:** 1890-05

**Authors:** 


### Editorial.

### WHAT SHOULD BE DONE WITH THE CRIMINAL INSANE.

It is not our purpose to offer an essay on the subject of insanity, either in general or on any special line, touching causes, effects or treatment. This will be left to experts and specialists. Our object is only to present a few reflections in reference to the *Criminal Insane.* We want to show how the interests of humanity can be subserved with the greatest safety to the public good—what is vulgarly called the "insane dodge" has so frequently cleared the guilty, that the people look with suspicion upon a plea of insanity, wherever or however righteously made, and are ready to cry out against it. The people are not the proper judges in such cases, nevertheless they exercise their opinions to such an extent that it is hardly safe to enter the *plea* of *insanity* in the most justifiable instances. The public mind has grown morbid on the subject of executing criminals, and the cause of humanity has suffered and will continue to suffer until some healthy legislation is had, which will restore public confidence in the courts. It is a shame and disgrace to penitentiary, to hang an insane individual. What shall we do with these unfortunate man slayers? In the first place, let the legislature enact laws requiring the individual to be tried before a commission of experts for lunacy, and if convicted, have him commuted to the State hospital for the insane, there to remain during natural life, not subject to executive clemency for release, unless so ordered by a majority vote of the members of each branch of the general assembly, for two consecutive terms. This, we believe, is the plan that should be adopted. It is merciful, it is just, and will do much towards appeasing the thirst of the people for blood. It will stay the hand of the mob—in fact it will refine, elevate, christianize and build up a higher civilization.

Many villains have escaped the punishment due them. Witnesses have been suborned, juries packed, and other wicked measures adopted to clear the guilty. This is no reason why mercy should be withheld from the poor unfortunate whose mind wanders over the vast territory of a diseased imagination whose impressions are morbid, whose impulses are unnatural, and whose passions run riot without the restraint of the reasoning faculties. Such a man is not morally, and should not be held legally responsible for his acts, but it is right that he should be sent as early as possible to an institution where he might be healed of his malady, and become a good citizen. Yes, send him before he commits any act of violence, before his case becomes incurable. You will thus protect him and the public. But if you wait until he has committed some terrible deed, try him for lunacy by a just, competent and wise commission, and if the allegation is sustained, commit him to the Asylum for the Insane for life time.

---

DR. LOUIS H. JONES.—Dr. Louis H. Jones, of this city, has been elected Prof. of Chemistry in the Atlanta Medical College, in place of Dr. H. P. Cooper, who resigned. The faculty could not have made a better selection.

---

No organizations in the United States have multiplied more rapidly in the past ten years than the sick-benefit, funeral-aid, death-benefit, and other kindred societies.

As they are generally confined to those who are in the humbler walks of life, the good they have done is incalculable, carrying substantial aid to thousands of stricken families and inspiring those who are fortunate enough in being members with a courage which might not exist in their hearts without them.

The members of these organizations will be glad to learn that Hon. Robert P. Porter, Superintendent of the Eleventh Census, will endeavor to secure the statistics of the noble work these associations are doing, and it is safe to say that no other branch of the census will be more interesting.

The business of gathering the data has been placed in charge of Mr. Charles A. Jenney, special agent of the insurance division, 58 William street, New York City, and all associations throughout the United States, whether incorporated or private, should assist by sending to him the address of their principal officers.

### THE ELEVENTH ANNUAL REPORT OF THE BOARD OF HEALTH OF THE CITY OF ATLANTA.

This will be found well worth reading by the physicians of this City, as well as all others, as it shows how healthy our city is, and how free from all forms of epidemic diseases.

It would be a good plan to scatter it through the country to help us build up our city.

### ABSTRACT OF REPORT ON OBSTETRICS AND GYNÆCOLOGY.

BY E. S. M'KEE, M. D., CINCINNATI, OHIO.

### ABORTION.

The treatment of abortion is a subject of great importance, because it is one which is always with us, and the careful handling of the case often saves the patient from long and troublesome as well as dangerous sickness. Of great interest to me is a case which happened recently in my practice. I was called to see a woman who was seven months pregnant with her third child. She was suffering from pains and seemed to be on the verge of aborting. I prescribed Dioviburnia made by the Dios Chemical Company, of St. Louis, in doses of a dessert-spoonful four times a day. The threatened abortion passed off and I was not again sent for until a month elapsed, when I found her in the same condition as before, suffering very much pain. She begged me for the medicine which had done her so much good on a former occasion, which I gave her in the same dose with a like result. On delivering her at full term of a fine boy, she volunteered the confession that she had, on both occasions mentioned, made desperate efforts to produce an abortion, and only sent for me when her sufferings became unbearable. I have also had marked results from this remedy in other cases, but the one here presented is of the most interest. I shall continue its use further.

